# A toddler with phylloid-type pigmentary mosaicism and ambiguous genitalia resulting from trisomy 14 induced by a der(Y)t(Y;14)

**DOI:** 10.1038/s41439-020-00113-x

**Published:** 2020-09-25

**Authors:** V. I. Romero, J. C. Pozo, S. Saenz, A. Llamos-Paneque, T. Liehr, K. Hosomichi, A. Tajima

**Affiliations:** 1 School of Medicine, Universidad San Francisco de Quito, Quito, Ecuador; 2 Specialty Army Hospital No. 1. Medical Genetic Services, Sciences of Life Faculty, School of Dentistry, International University of Ecuador, Quito, Ecuador; 3 Institute of Human Genetics, Jena University Hospital, Friedrich Schiller University, Am Klinikum 1, D-07747 Jena, Germany; 4 Department of Bioinformatics and Genomics, Kanazawa University, Kanazawa, Japan

**Keywords:** Genetics research, Mosaicism, Genetic testing, Paediatrics

## Abstract

A 1-year-old baby with phylloid-type pigmentary mosaicism, hypotonia, ambiguous genitalia, and a positive screening test for congenital adrenal hyperplasia was referred. Previous sonograph, cytogenetics, and metabolic profile were inconclusive, therefore we performed an additional karyotype and a molecular cytogenetics studies. A mosaic karyotype 45,X/46,X,der(Y)t(Y;14) was characterized in peripheral blood. Congenital adrenal hyperplasia genes were sequenced and the results were negative. The ambiguous genitalia was the result of the special gonosomal mosaicism. The low level of trisomy 14 led to minor physical characteristics and mild mental retardation; also, Turner syndrome features can be expected rather than severe trisomy 14 stigmata.

## Introduction

Trisomy 14 is one of the aneusomies that is compatible with live if present in mosaic. It has been reported in ~50 patients, most of them with proximal or distal trisomy of chromosome 14^1^. Clinical presentation is variable, depending on the degree of mosaicism, size of the trisomic segment, concurrence with other chromosomal imbalances, and the parental origin of the rearrangement due to the possible imprinting effects. However, predominant symptoms are prenatal and postnatal growth failure, ear abnormalities, congenital heart defects, developmental delay, and genitourinary abnormalities^2,3^, as well as narrow palpebral fissures, broad nose, dysplastic and/or low set, micrognathia, and short neck^4^.

Here, we report a patient with ambiguous genitalia and pigmentary mosaicism with a unique karyotype. This case is relevant because it has two uncommon cytogenetics events: an almost complete trisomy of chromosome 14, due to a Y-autosome translocation, and a mosaic for Turner syndrome.

## Patient

**Fig. 1:**
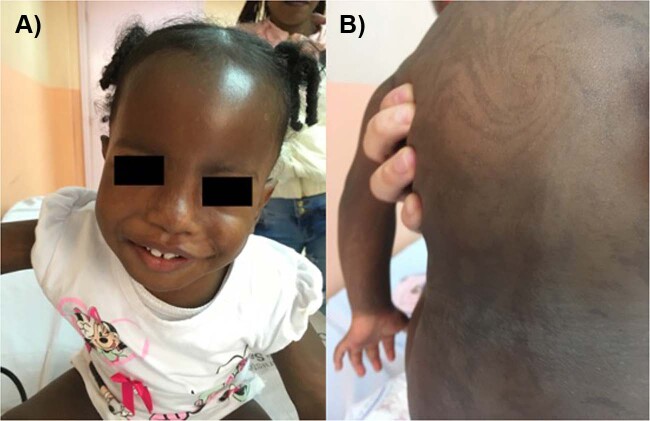
Patient’s physical characteristics. **a** Facial asymmetry. **b** Phylloid-type hyperpigmentation.

A 1-year-old patient was referred due to growth retardation and mild global developmental delay, hypotonia, pigmentary mosaicism with phylloid hyperpigmentation along the body, and ambiguous genitalia (enlarged phallus, with a single urogenital sinus and almost complete fusion of the labia, Prader scale type III) with no visible testicles. The child was born by vaginal delivery at 38.6 week from nonconsanguineous parents, a 19-year-old mother and 20-year-old father. The birth weight was 2580 g (4.5% percentile), length was 46 cm (2% percentile), and cephalic circumference 32.7 cm (8.2% percentile). Physical examination revealed unilateral left palpebral ptosis, broad nose and forehead, and hypochromic cutaneous spots on the neck, (Fig. 1a, b). In addition, the neonatal screening was positive for congenital adrenal hyperplasia and adequate medication was started immediately.

The initial workflow focused on the sex characterization during the first year; however, the results of various tests were inconclusive. Results that suggested a female phenotype were: an initial peripheral blood karyotype reported as 45,X[80]/46,XX[20] molecular test showed an absence of the *SRY* gene, and an initial pelvic ultrasound revealed a vaginal channel, clitoral hypertrophy, and bilateral inguinal hernias. On the other hand, results that suggested a male phenotype were a subsequent computed tomography revealing the presence of a right testicle in the scrotum, a missing left testicle, and bilateral ureteral reflux with mild dilation (incidental finding) and a second pelvic sonography describing testicles in the inguinal duct. All of the gonad phenotype characterization was obtained only by imaging tests and not by biopsy due to the patient’s young age.

For clarification, a second peripheral blood sample was taken. Karyotype was determined by banding cytogenetics and fluorescence in situ hybridization (FISH). The karyotype from blood revealed following result: 45,X[24]/46,X,der(Y)t(Y;14)(p11.32;q12)^4^ and FISH test confirmed the presence of the *SRY gene* and *AZF* region being intact. In FISH, a whole chromosome painting probe for chromosome 14 (wcp 14) and probe for all acrocentric p-arms (midi54) together with and a probe specific for *SRY* gene (Vysis, USA) were applied (Supplementary Fig. 1). Parental cytogenetic studies did not find any abnormalities in 20 metaphases.

FISH analysis reported that the chromosome 14 region from the derivative chromosome does not include the imprinted genes, therefore the patient does not have clinical characteristics from UPD (Supplementary Fig. 1). To identify the specific genes within the junction region, we performed whole-genome sequencing with high depth (×60). The reads from the junction region did not map the reference from chromosome 14 or Y. The junction region could not be identified either due to low percentage of the derivative chromosome cell line or an unknown region located in the junction.

The ambiguous genitalia could be the consequence of either the chromosomal aneuploidy or the congenital suprarenal hyperplasia. The first screening for congenital suprarenal hyperplasia was positive, the second screening was negative, and a confirmatory test was inconclusive, however prednisone treatment was started. After referral, sequencing was performed to corroborate a pathogenic variation on genes associated to congenital suprarenal hyperplasia. No genes, including *CYP21A2*, *CA21H*, *HSD3B3*, *CYP11B1*, *P450C11*, *FHI*, *POR*, CYP17A1, and *STAR*, obtained by whole-genome sequencing were reported as pathogenic; therefore, prednisone tapering was started to avoid steroids side effects.

## Discussion

The here reported patient had a complex sex characterization with a trisomy 14 with a derivate Y chromosome and Turner syndrome mosaic. To our knowledge, there is no previous similar report.

One of the longest series in which cases with pure partial trisomy 14 are reported, includes 51 patients, and in none of them did the trisomy have a fairly large segment of chromosome 14 as presented in this case^1^ (Supplementary Fig. 2).

One mosaic of Turner and trisomy 14 was reported describing an individual with swirly areas of hyperpigmentation, short stature, broad nasal bridge, mouth with downturned corners, short and wide neck, and body asymmetry secondary to right hemihyperplasia^5^.

Our patient shares similarities of the swirly areas of hyperpigmentation with mosaic of trisomy 14 and with Turner syndrome, in addition to a global developmental delayed found in most of the cases mentioned above (Fig. 1a, b)^2,5–8^. However, this patient does not have a cardiac condition, abdominal, limbs or rib deformities, or behavioral problems such as impulsivity, obstinacy, and compulsive skin picking (Table 1). The probable mechanism causing the 45,X/46,X,der(Y)t(Y;14)(p11.32;q12) is a de novo translocation in the paternal germline between chromosomes 14 and Y. During meiosis, the spermatozoid produced by the adjacent segregation of the abnormal germline, fertilized a normal oocyte resulting on a 46,X,der(Y)t(Y;14)(p11.32;q12) fertilized egg with a subsequent loss of the additional derivative chromosome 14 from the trisomic cell during mitotic division on the early cell divisions. The loss of the additional chromosome resulted on the 45,X cell line, due to trisomy rescue, limited the clinical impact of a uniparental disomy of chromosome 14 (Supplementary Fig. 3).

**Table 1:** Physical characteristics of trisomy 14 variations.

	Trisomy 14	Trisomy 14 mosaicism + UDP mat	Trisomy 14 mosaicism + UDP pat	Trisomy 14 der 5	Trisomy 14 der 9	Trisomy 14 der 12	Trisomy 14 + Turner	Our patient
Developmental status								
Global developmental delay	+	+		+	+	+	+	+
Skin								
Hypopigmentation		+			+			
Pigmentary mosaicism	+	+					+	+
Craniofacial appearance								
Microcephaly	+	+		+		+	+	
Macrocephaly								
Prominent forehead	+	+	+					+
Hypertelorism	+	+						
Coloboma								
Short neck	+		+				+	+
Ear anomalies	+					+		
Broad nose	+	+				+	+	+
High arched palate	+	+				+		
Micrognathia	+	+	+	+				
Cleft lip or palate	+			+				
Irregular teeth					+			
Growth and maturation								
Height								
Normal								
Abnormal	+	+		+	+	+	+	+
Weight								
Normal								
Abnormal	+	+		+	+	+	+	+
Other findings								
Pulmonary					+	+		
Cardiologic	+		+	+		+		
Chest		+	+				+	
Abdominal		+	+	+				
Renal				+				
Suprarrenal hyperplasia								
Genitals	+	+		+				
Ambiguous genitalia								+
Arms and legs	+		+				+	
Fingers	+	+			+		+	
Toes	+	+		+	+		+	

Regarding the ambiguous genitalia, the Y-autosome translocation identified by karyotyping, included genes that contribute to a male phenotype, *SRY*, and all region from *DAZ1* to *DAZ4*; however, gonadal histologic exploration is still pending. The whole-genome gene sequencing analysis denied that a congenital adrenal hyperplasia caused the ambiguous genitalia and was helpful on the decision of discontinuing the steroid treatment.

In order to determine the precise gene content of the trisomic region and the specific location of the chromosome breakage, the use of a more sophisticated cytogenetic molecular technique like array comparative genomic hybridization is necessary; however, this technology was not available. The prognosis for the patient most likely resembles individuals with Turner syndrome rather than a trisomy 14 mosaicism or cases with UPD.

Therefore, since the patient is still very young, the clinic follow-up is compulsory to improve the phenotypic delineation and to decide on any surgical decisions regarding the internal gonads.

## Supplementary information

Supplementary Material 1

Supplemental Material 2

Supplemental Material 3
