# Supplementary figures and images for: A toddler with phylloid-type pigmentary mosaicism and ambiguous genitalia resulting from trisomy 14 induced by a der(Y)t(Y;14)
Source: Hum Genome Var. 2020 Sep 25;7:28. doi: 10.1038/s41439-020-00113-x (PMC7519037; doi:10.1038/s41439-020-00113-x)

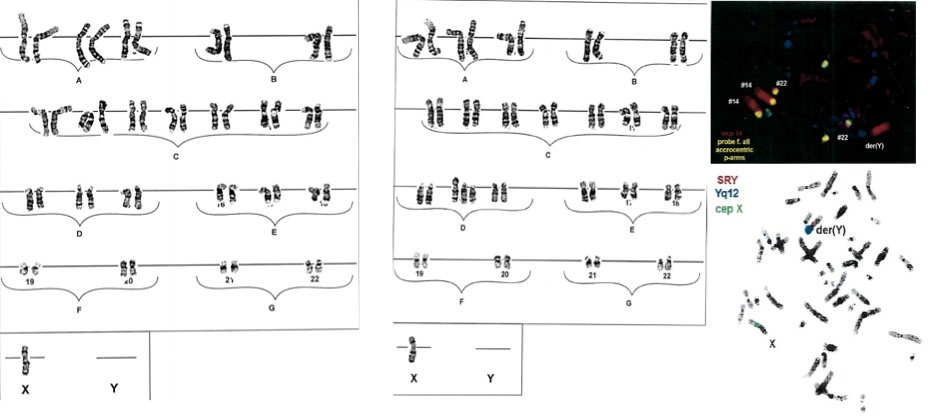

Supplement: Supplementary file 1 — Supplementary Material 1 [file 41439_2020_113_MOESM1_ESM.jpg]

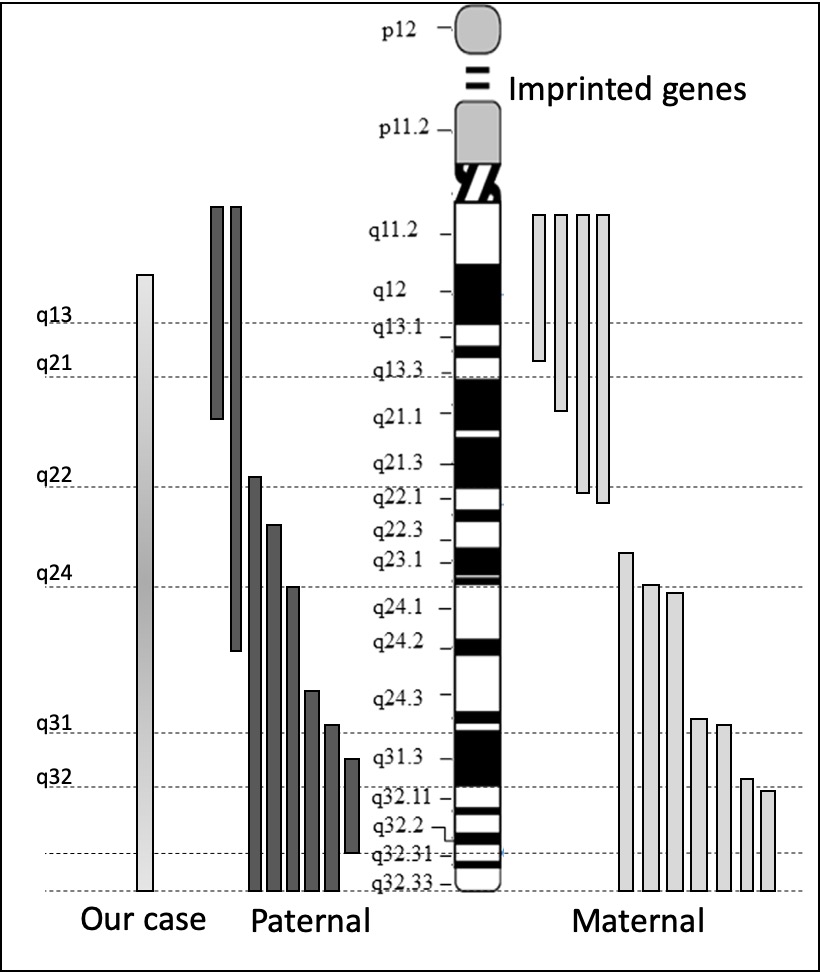

Supplement: Supplementary file 2 — Supplemental Material 2 [file 41439_2020_113_MOESM2_ESM.jpg]

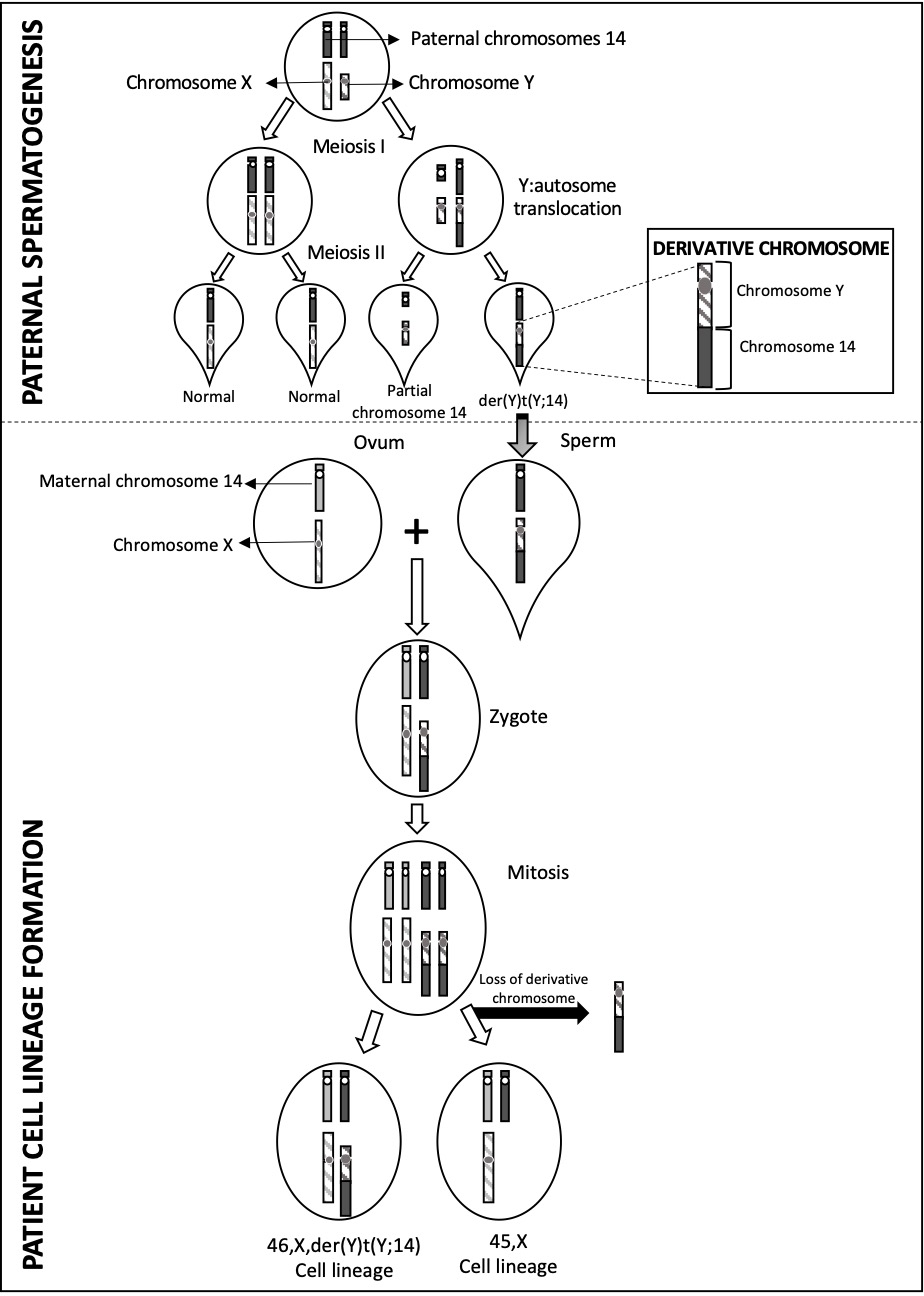

Supplement: Supplementary file 3 — Supplemental Material 3 [file 41439_2020_113_MOESM3_ESM.jpg]
